# Critical Role of the Sulfiredoxin-Peroxiredoxin IV Axis in Urethane-Induced Non-Small Cell Lung Cancer

**DOI:** 10.3390/antiox12020367

**Published:** 2023-02-03

**Authors:** Yanning Hao, Hong Jiang, Pratik Thapa, Na Ding, Aziza Alshahrani, Junichi Fujii, Michel B. Toledano, Qiou Wei

**Affiliations:** 1 Department of Toxicology and Cancer Biology, University of Kentucky, Lexington, KY 40506, USA; 2 Department of Biochemistry and Molecular Biology, Graduate School of Medical Science, Yamagata University, Yamagata 990-8560, Japan; 3 Institute for Integrative Biology of the Cell (I2BC), CEA, CNRS, Université Paris-Saclay, 91198 Gif-sur-Yvette, France; 4 Markey Cancer Center, University of Kentucky, Lexington, KY 40536, USA

**Keywords:** sulfiredoxin, peroxiredoxin, lung cancer, tumorigenesis

## Abstract

Non-small cell lung cancer (NSCLC), the most common type of lung cancer, etiologically associates with tobacco smoking which mechanistically contributes to oxidative stress to facilitate the occurrence of mutations, oncogenic transformation and aberrantly activated signaling pathways. Our previous reports suggested an essential role of Sulfiredoxin (Srx) in promoting the development of lung cancer in humans, and was causally related to Peroxiredoxin IV (Prx4), the major downstream substrate and mediator of Srx-enhanced signaling. To further explore the role of the Srx-Prx4 axis in de novo lung tumorigenesis, we established Prx4^−/−^ and Srx^−/−^/Prx4^−/−^ mice in pure FVB/N background. Together with wild-type litter mates, these mice were exposed to carcinogenic urethane and the development of lung tumorigenesis was evaluated. We found that disruption of the Srx-Prx4 axis, either through knockout of Srx/Prx4 alone or together, led to a reduced number and size of lung tumors in mice. Immunohistological studies found that loss of Srx/Prx4 led to reduced rate of cell proliferation and less intratumoral macrophage infiltration. Mechanistically, we found that exposure to urethane increased the levels of reactive oxygen species, activated the expression of and Prx4 in normal lung epithelial cells, while knockout of Prx4 inhibited urethane-induced cell transformation. Moreover, bioinformatics analysis found that the Srx-Prx4 axis is activated in many human cancers, and their increased expression is tightly correlated with poor prognosis in NSCLC patients.

## 1. Introduction

The United States is expected to see 1,918,030 new cancer cases and 609,360 cancer deaths by 2022, with 350 deaths per day due to lung cancer, the major cause of cancer death [1]. The mortality of lung cancer in recent years shows a trend of decline due to improved medical practice responsible for approximately 81.3% of lung cancer fatalities, with an additional 2.8% of deaths occurring as a result of exposure to second-hand smoke [2]. Cigarette smoking exposes airways to a variety of chemical elements and irritating particles that accumulate and contribute to chronic pulmonary inflammation or other pathological events, including cancer [3]. Copious amounts of reactive oxygen and nitrogen species (ROS and RNS) contained in and induced by cigarette smoke play a significant role in pulmonary inflammation and LUAD by increasing oxidative stress and DNA damage [4,5]. The production of ROS within cells maintains homeostasis with a number of endogenous antioxidants, such as peroxiredoxins (Prxs). Given that the oxidative stress caused by the imbalance of ROS/antioxidants exhibits a double-edged effect on biological activities in both normal and cancer cells, and is tightly related to initiating tumorigenesis, promoting proliferation, as well as supporting metastasis [6,7]. The mechanism and potential therapeutical target of this ROS/antioxidants’ imbalance in LUAD have not been critically studied. Understanding the connection between cell response to this imbalance is advantageous for the establishment of new strategies for lung cancer prevention.

Peroxiredoxin (Prx) has been identified as an essential member of the antioxidant family containing reactive cysteine (Cys) residues, and it functions by attenuating oxidative stress and regulating redox signaling in mammalian cells by eliminating H_2_O_2_ [8]. In humans, six members of the Prx family have been characterized (Prx1-6) [9]. Growing evidence suggests the involvement of Prxs in cancer progression, including cell proliferation [10], apoptosis [11], metastasis [12], and radiation resistance [13]. Of the six Prxs, Prx4 is highly expressed in a variety of organs and is the only secretory isoform [14]. Prx4 has a unique hydrophobic N-terminus, which serves as a signal sequence in charge of the endoplasmic reticulum location and secretion to extracellular regions. Secreted Prx4 in the reduced form can protect blood vessels from extracellular ROS via interaction with heparin sulfate and binding to endothelial cells [14]. Prx4 also has a critical role in regulating physiological activities such as differentiation, apoptosis, embryonic development, lipid metabolism, and immunological response [15]. Numerous studies have raised the possibility that Prx4 acts as a biological marker for a variety of diseases, including sepsis [16], type 2 diabetes [17], fatty liver [18], Alzheimer’s Disease [19], and atherosclerosis [20]. However, what interests us more are the as yet-unclear roles that Prx4 plays in malignancies. The connection between this antioxidant and carcinogenesis has received a lot of attention since it was recently discovered that Prx4 is overexpressed in a number of human cancers. Numerous researchers have demonstrated that Prx4 promotes tumor development, spread, and recurrence, as well as resistance to treatment. For instance, Prx4 is upregulated and correlated with various clinicopathological parameters in prostate cancer [21]. In lung cancer, the overexpression of Prx4 promotes lung tumorigenesis in vivo through the phosphorylation of c-Jun and nuclear factor-*κ*B [22]. To maintain the intracellular antioxidant function, Prx4 needs to be reduced by Sulfiredoxin (Srx), which serves as a toggle to activate Prx4 continually. Among all 2-Cys containing Prxs, Prx4 has the highest binding affinity with Srx [23,24]. Srx has been suggested as playing an oncogenic role in the development of lung cancer in smokers. Previously, we demonstrated that the silencing of Srx led to a lower number and smaller size of lung tumors through inhibition of tumor cell proliferation while increasing tumor cell apoptosis in vivo [25]. The primary function of the Srx-Prx4 axis is to maintain the redox balance by mediating oxidative signaling that contributes to the overall cellular response to ROS [26]. Our previous study further demonstrated that the Srx-Prx4 axis contributes considerably to the maintenance of anchorage-independent colony growth, cell motility, and invasion by the activation of the RAS-MEK-ERK pathway in lung cancer cells [24]. Based on these and other studies, we believe that the Srx-Prx4 axis may play a critical role in lung tumorigenesis and cancer development.

Among all tobacco components, urethane was initially identified as a carcinogen in 1943 [27]. Since then, it has been used extensively in experimental mouse models to study the process of lung cancer development [28]. In this model, lung tumors are commonly recognized as LUAD, and tumor cells are thought to arise from Clara or type II alveolar epithelial cells [29]. The gene expression profiling of urethane-induced mouse lung tumor cells are similar to those identified in human LUAD [30], further supporting that pulmonary adenocarcinoma induced by urethane in mice reflects similar histopathology of LUAD in human, which can be used to study fundamental mechanisms of lung tumorigenesis. To understand the contribution and mechanism of the Srx-Prx4 axis in lung cancer, we developed Prx4 knockout (Prx4^−/−^), Srx/Prx4 double knockout (Srx^−/−^/Prx4^−/−^, DKO) mice. Together with wild-type litter mates, these mice were evaluated for urethane-induced lung tumor development. Findings from this study will provide novel insights in the role of the Srx-Prx4 axis in lung cancer development, and exhibit a promising target for cancer treatment.

## 2. Materials and Methods

### 2.1. Establishment of Prx4^−/−^ Mice and Prx4^−/−^/Srx^−/−^ Mice

All schemes of mouse breeding and experimental protocols were reviewed and approved by the University of Kentucky Institutional Animal Care and Use Committee (UK protocol 2016–2306). All procedures on mice were conducted following the Policy on Humane Care and Use of Laboratory Animals, and the Guidelines of the Animal Care and Laboratory Animal Welfare (NIH). Mice, regardless of strain or genetic background, were all housed in standard cages in temperature-controlled environments under a 12-h light/12-h dark cycle with ad libitum access to standard chow and water unless otherwise indicated. Original Prx4^−/−^ mice in the C57BL/6 background were originally established by Iuchi et al. [31]. Prx4^−/−^ mice in pure FVB/N background were further established by cross breeding the C57BL/6 mice with FVB/N mice for multiple generations (>10). To obtain Prx4^−/−^ female mice, Prx4 genomic DNA was cloned. In the first intron of Prx4, the phosphoglycerate kinase-driven neomycin cassette flanked by loxP sequences was inserted followed by an extra loxP sequence and introduced upstream of the first exon. Prx4^−/y^ male mice were generated by breeding Prx4^flox/+^ female mice with male mice who express Cre recombinase as described before [31]. Srx^−/−^ C57BL/6 mice [32], were first bred with FVB/N mice and after multiple generations (≥10) of backcross breeding, the offspring Srx^−/−^ FVB mice were used to breed with Prx4^−/−^ mice to generate Prx4/Srx double knockout (DKO) mice. The genotype of KO and DKO mice was confirmed by PCR-based mouse genotyping as described previously. 

### 2.2. Urethane-Induced Mouse Lung Cancer Protocol and Tissue Processing

A randomized, double-blind experimental design was used to eliminate any potential subjective bias. Wildtype (WT), Prx4^−/−^ (KO), and Prx4^−/−^/Srx^−/−^ (DKO) mice were given an intraperitoneal (i.p.) injection of urethane (U2500, Sigma, St. Louis, MO, USA) at 1g/kg body weight dissolved in saline at 8 weeks of age once a week for three weeks continuously as described in a previous report [25]. After the injections, all mice were fed a normal diet and water for an additional 9 weeks. The body weight of mice was monitored during the whole experimental period. After 12 weeks, all mice were sacrificed, and the lungs were perfused with PBS and extracted, then fixed in paraformaldehyde and stored in 70% ethanol. Tumors on the lungs’ surface were examined and measured with a digital caliper. After standard paraffin embedding and sectioning to visualize microscopic tumors, hematoxylin and eosin (H&E) stained slides were used to count the number and size of tumors under a dissecting scope or by low-magnification microscopy. Tissue slides were also used for immunohistochemical staining (IHC). For IHC, paraffin-embedded tissue slides were subjected to dewaxing and rehydration, then stained for Prx4 (1:500, ab184167, Abcam, Cambridge, UK), Srx, Ki67 (1:40, ab16667, Abcam), F4/80 (1:50, sc-377009, Santa Cruz, Dallas, TX, USA), CD86 (1:50, sc-28347, Santa Cruz), or CD163 (1:50, sc-33715, Santa Cruz), following the standard protocol of ABC kit (PK-4001, Vector Laboratories, Newark, CA, USA. Then, these slides were counterstained with hematoxylin (H3403, Vector Laboratories), and coverslips affixed by DPX mountant (06522, Sigma). Quantification was performed by Imagescope Algorithm analysis software (Aperio Technology, CA, USA).

### 2.3. Cell Culture and Establishment of Stable Knockdown Cells

BEAS2B (normal human bronchial epithelial cell line) and HEK293T (human embryonic kidney cell line) cells were commercially obtained (ATCC, Manassas, VA, USA). BEAS2B cells were cultured in Bronchial/Trachea Epithelial Cell Growth Medium, and HEK293T cells were cultured in the Dulbecco’s Modified Eagle’s medium supplemented with 10% fetal bovine serum, 50 U/mL penicillin-streptomycin, and 25 ug/mL gentamicin.

ShRNA-based knockdown studies were conceived and carried out strictly in accordance with previously published protocol [33]. All shRNA constructs, including the MISSION^®^ pLKO.1-puro control vector, MISSION^®^ Non-Target shRNA (shNT), and shRNAs targeting Prx4 (shPrx4) or Srx (shSrx), were obtained commercially from Sigma Aldrich. Lentiviral particles expressing shRNAs were created in HEK293T cells in accordance with the manufacturer’s recommended transfection and virus production protocols. A549 cells were infected with lentiviral particles containing either control shNT or shRNA targeting the coding region of Prx4 or Srx and maintained in the puromycin-containing medium (1.0 µg/mL).

### 2.4. Intracellular ROS Level Measurement

The intracellular ROS level was detected using the DCFDA method [34]. DCFDA is a cell-permeable non-fluorescent probe, but in the presence of ROS, this reagent is oxidized to the highly fluorescent 2′,7′-dichlorofluorescein. Briefly, cells were seeded in a 96-well plate at 2 × 10^4^ cells/well and treated with different concentrations of urethane. We used cells treated with 100 μM H_2_O_2_ for 30 min as the positive control. After 48 h of treatment, the cells were incubated with DCF-DA 25 μM for 45 min, and the fluorescence of 2′,7′-dichlorofluorescein was detected using Cytation 5. The fold change of intracellular ROS level was calculated using the following equation: (F_test_ − F_blank_)/(F_control_ − F_blank_), where F_test_, F_control,_ and F_blank_ represent the fluorescence readings from the urethane-treated cells, no treatment cells, and no stained cells, respectively.

### 2.5. Immunoblotting

After trypsinization, cells were harvested and resuspended in radio-immunoprecipitation assay buffer with 1% protease inhibitor cocktail (Santa Cruz Biotech, Dallas, TX, USA). Western blotting was performed following the standard protocol. Protein samples were separated using SDS/PAGE at a gradient concentration of 4–12% and electroblotted onto PVDF membranes. After blocking with 5% nonfat milk in Tris-buffered saline (20 mM Tris/HCl, pH 7.6, and 150 mM NaCl), the blots were incubated overnight at 4 °C with either Prx4, Srx, or actin diluted in BSA. The secondary antibody conjugated to HRP was applied, and the signal was detected using chemiluminescence utilizing ECL followed by exposure to long machine. 

### 2.6. Bioinformatics Analysis

ATGC and GEO datasets were used to evaluate the expression level of Srx and Prx4 in lung adenocarcinoma and explore Prx4 in lung cancer prognostic through survival analysis.

#### 2.6.1. Data Source

LUAD-related RNA sequencing (RNA-seq) data (510 LUAD: 58 normal), methylation data (455 LUAD: 32 normal), and copy data (514 LUAD) were acquired from the Cancer Genome Atlas (TCGA) database (https://portal.gdc.cancer.gov (accessed on 5 May 2022)), and 497 LUAD samples with survival and clinical information in TCGA-LUAD. Moreover, we also acquired the GSE19804, GSE27262, GSE31210, and GSE30219 datasets from the Gene Expression Omnibus (GEO) database (https://www.ncbi.nlm.nih.gov/ (accessed on 5 May 2022)), of which the GSE19804 dataset contains 60 LUAD and 60 normal samples, and the GSE27262 dataset contains 25 LUAD and 25 normal samples. In addition, the GSE31210 and GSE30219 datasets contain 226 and 293 LUAD samples with survival information, respectively.

#### 2.6.2. Expression Analysis of Prx4

The expression of Prx4 in all samples was extracted from the TCGA dataset, and we compared the expression of Prx4 in LUAD and normal samples using the Wilcoxon test method. The abundance of Prx4 was further validated in the GSE19804 and GSE27262 datasets. To investigate the protein abundance of Prx4 in LUAD, the example of the immunohistochemical results in LUAD and normal tissues were obtained from the human protein atlas (THPA) database (Human Protein Atlas, https://www.proteinatlas.org/ (accessed on 31 January 2023)) [35].

#### 2.6.3. Clinical Correlation Analysis

The 497 LUAD patients with clinical information in TCGA-LUAD were classified into Prx4 high and Prx4 low groups based on the optimal threshold of Prx4 expression. Differences in overall survival (OS) between the Prx4 high/low groups were analyzed by the “survival” R package. In addition, the difference in OS of Prx4 was further validated in the GSE31210 and GSE30219 datasets. Based on the different clinicopathological characteristics (age; gender; pathologic T, N, M; tumor stage; tissue or origin tobacco smoking history) of LUAD patients in the TCGA dataset, Prx4 expression of different clinical features was plotted using the “ggplot2” R package. Additionally, the differences in different clinical features for the high and low PRX4 groups were analyzed by chi-square test.

#### 2.6.4. Functional Enrichment Analysis of PRX4

To reveal the function of Prx4 in LUAD, the genes associated with Prx4 were analyzed using “LinkFinder” in the LinkedOmics database, and “pheatmap” R package to plot the heat map of genes significantly positively and negatively associated with Prx4. Then, the gene set enrichment analysis (GSEA) functional enrichment analysis of Prx4 was executed by the LinkedOmics database, and the top 25 descriptions were selected according to the enrichment score positive and negative rankings for visualization.

#### 2.6.5. Immune Infiltration Analysis

The immune response is important in tumor growth and metastasis [36]. To investigate the immune infiltration in LUAD and normal samples from the TCGA dataset, the ratio of 22 immune cells in each sample was obtained by the CIBERSORT algorithm, and samples with *p* > 0.05 were excluded. A heat map for the scores of the 22 immune cells was then drawn based on the scores of each immune cell, and the immune cells were compared for LUAD and normal samples by the Wilcoxon test method. Moreover, the expression of the 22 immune cells in the Prx4 high/low groups was compared by the Wilcoxon test. The differential immune cells from the above two groups were intersected to obtain the common differential immune cells. Correlations between Prx4 and common differential immune cells was calculated using Spearman method. We also analyzed the correlation of Prx4 with immunomodulators (immunostimulants and immunosuppressants) using the TISIDB database (http://cis.hku.hk/TISIDB/index.php (accessed on 1 June 2022)). Finally, in order to understand whether there were differences in macrophages in Prx4 high/low groups, we performed immune cell infiltration analysis in patients of the Prx4 high/low groups by “GSVA” R package [37], “xcell” R package [38], and “immundeconv” R package [39] using single sample gene set enrichment analysis (ssGSEA), xcell, and EPIC methods, respectively, and then screened for differential immune cells between Prx4 high/low groups by Wilcoxon test method.

#### 2.6.6. Investigation of the Bio-Mechanisms of Aberrant Prx4 Expression

We analyzed the Prx4 gene by different histological dimensions such as mutation, methylation, and copy number to further examine the biologic mechanism of Prx4 expression abnormalities. First, the genetic mutation frequency and mutation characteristics of Prx4 in LUAD were analyzed by the CbioPortal database (http://cbioportal.org (accessed on 27 January 2023)) [40,41]. Then, the methylation sites corresponding to Prx4 were obtained from the TCGA database, and the differences between LUAD and normal samples were analyzed using the “limma” R package [42]. Next, the correlation between Prx4 expression level and its corresponding methylation site was calculated by Spearman method. The copy number variation data of LUAD were obtained from the TCGA database to further obtain the copy number variation status of Prx4. The miRNA prediction of Prx4 was performed by the miRwalk database (v3.0 http://mirwalk.umm.uni-heidelberg.de/ (accessed on 6 June 2022)), while the ENCORI database (https://starbase.sysu.edu.cn/index.php (accessed on 6 June 2022)) was used for miRNA prediction. The threshold values were set as CLIP-Data ≥ 1, Degradome-Data ≥ 0, pan-Cancer ≥ 0, programNum ≥ 1. Then, the predicted results of the two databases were taken as a concatenation to obtain miRNA-mRNA regulatory relationship pairs, and the network was constructed by Cytoscape software. Then, the miRNA expression data of LUAD were acquired from the TCGA database and matched with the predicted miRNAs above. After that, the matched miRNAs were analyzed by univariate cox regression, and the *p* was set to 0.05 to obtain the miRNAs. The miRNA expressions were extracted from the miRNA expression matrix, and the 497 LUAD samples were classified into an miRNA high and low expression group on the basis of the mean of miRNA expressions. The Kaplan-Meier (K-M) curve was used to analyze the OS of miRNA high and low expression groups.

### 2.7. Statistical Evaluation

All data were expressed as mean ± SD of three independent experiments or as specified. Statistical analyses were performed using Prism 8 (GraphPad Software, San Diego, CA, USA). Statistical significances between groups were determined based on a two-tail Student’s *t*-test or ANOVA, and a *p*-value of less than 0.05 was considered to be statistically significant (*, *p* < 0.05; ns, *p* > 0.05).

## 3. Results

### 3.1. Loss of Prx4 or Srx/Prx4 Inhibits Tumor Formation and Cell Proliferation

**Figure 1:**
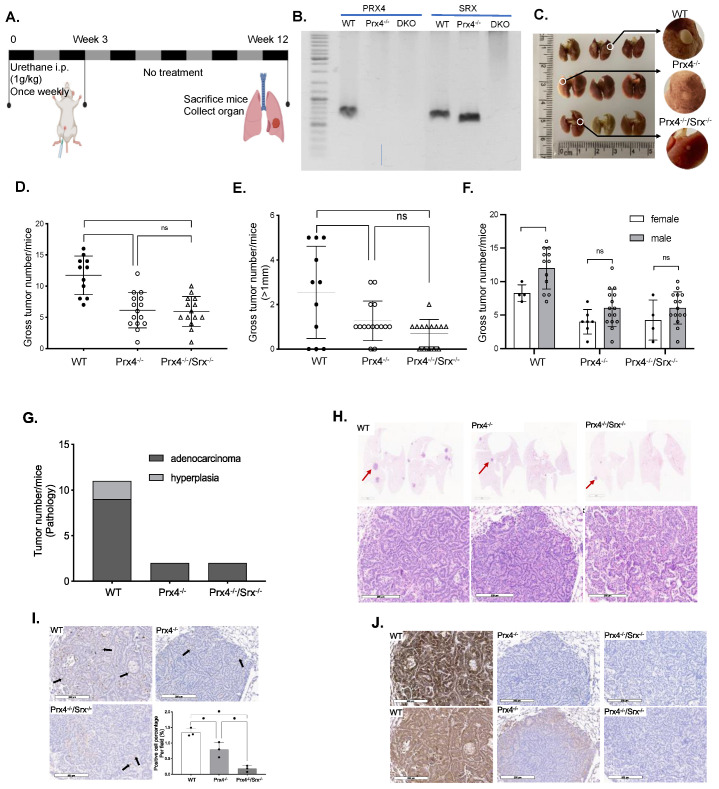
Loss of Prx4 or Srx/Prx4 suppresses tumor formation and cell proliferation in the urethane-induced lung adenocarcinoma mouse model. (**A**) Schematic illustration of the methodology for 12-week urethane-induced lung tumorigenesis in mice. (**B**) Genotype verification. (**C**) Lung gross images with zoom-in of tumors on the surface. (**D**) Comparing the average tumor number of each mouse with three genotypes (>0.2 mm). (**E**) Comparing the average tumor number of each mouse with three genotypes (>1 mm). (**F**) Comparing the average tumor number of each mouse with gender difference for the three genotypes (>0.2 mm). (**G**) Pathologic diagnosis of tumors for the three genotypes based on H&E histology. (**H**) H&E staining of lung tumors microscopic image for the three genotypes. Arrows identify examples of tumor. (**I**) Anti-Ki67 staining of tumor tissue from three genotypes and quantitative analysis of staining intensity. Arrows identify Ki-67 positively stained cells (brown). (**J**) Anti-Prx4 (upper) and -Srx (lower) staining of tumor tissue from three genotypes. (*, *p* < 0.05; ns, *p* > 0.05).

To study the biological role of the Srx-Prx4 axis in lung cancer, a well-established urethane-induced lung tumor protocol was used on all mice. Briefly, mice were given three doses of urethane through intraperitoneal injection and sacrificed after 12 weeks (Figure 1A). It has been shown that the susceptibility of mice to urethane may differ with strain and sex [43]. Therefore, all mice in our studies were developed in pure FVB/N background, and both males and females were used in each group. Previously, we found that Srx knockout mice were resistant to urethane-induced lung tumorigenesis [25]. In this study, we focused on whether the loss of Prx4 alone or the absence of both Srx and Prx4 had similar effects on mouse lung tumorigenesis. All mice, either Srx/Prx4 knockout alone or DKO, were viable, healthy, fertile, and without any developmental defects in laboratory conditions. Mouse genotypes were confirmed by using primers specific to amplify target genes within the *PRDX4* or *SRXN* locus (Figure 1B). All mice were able to survive to the end of the experimental period, and mouse lungs were extracted after euthanizing. Macroscopically, urethane-induced tumors were mainly manifested as white, solid nodules in the surface or periphery of the lung, and tumors found in wild-type mice were generally larger in size and tended to cluster together compared with either Prx4^−/−^ or DKO (Figure 1C). All mice, regardless of phenotype, developed tumors in the lung, thus there was no difference in tumor incidence among the three groups. After counting the number of tumors (≥0.2 mm) and measuring their diameters, we found that the average number of tumors per mouse (tumor multiplicity) in either the Prx4^−/−^ or Prx4^−/−^/Srx^−/−^ group is significantly reduced (by around 40%) compared with that of the wild-type group (Figure 1D). Such a reduction was more evident in all knockout mice when the number of tumors with a diameter larger than 1.0 mm was compared (Figure 1E). However, there was no statistical significance in tumor multiplicity or size distribution when Prx4^−/−^ mice were compared with Prx4^−/−^/Srx^−/−^ mice. In terms of sex difference, the number of tumors in male mice was significantly higher than that of females within the wild-type group, and a similar trend was observed in knockout groups (Figure 1F). Next, fixed lungs from each group were randomly picked and tissue slides were obtained by sequential sectioning. H&E staining and histopathology analysis were performed. Nodules in the lung of wild-type mice were mainly diagnosed as well-differentiated lung adenocarcinoma, whereas similar masses in all knockout mice were categorized mainly as aberrant hyperplasia or early adenoma due to their localized foci or limited size (Figure 1G,H).

To understand why the loss of Prx4 or Prx4/Srx reduced urethane-induced tumor multiplicity and size, we examined the rate of cell proliferation in tumors from different genotypes. The nuclear protein Ki67 is widely applied as a proliferation marker and prognostic indicator for cancer evaluation in regular pathological examinations since its expression is closely correlated with tumor cell proliferation and growth. Mouse lungs from the three groups were stained for Ki67. Compared with normal lung tissue, there is an apparent increase in the cell proliferation rate in adenocarcinoma and hyperplasia as indicated by more and stronger Ki67^+^ staining. However, by comparing the staining intensity of Ki67^+^ in tumors from the three groups, cell proliferation rates in tumors from Prx4^−/−^ and Prx4^−/−^/Srx^−/−^ mice are much slower than from wild-type mice as indicated. Additionally, tumors in Prx4^−/−^/Srx^−/−^ mice have less positive Ki67 staining than in Prx4^−/−^ mice, which shows the lowest cell proliferation rate in Prx4^−/−^/Srx^−/−^ mice (Figure 1I). We also examined the expression level of Prx4 and Srx in mice lung tissue and tumors of each group. Positive staining of both proteins is much stronger in tumor tissue than in normal lung tissue in the WT group (Figure 1J). We also found that the Srx expression level was lower in Prx4^−/−^ group than in the WT group, which indicated that loss of Prx4 downregulated Srx expression in tumor tissue.

In summary, we found that genetic depletion of Prx4 or Prx4/Srx has no effect on tumor incidence of mice receiving urethane treatment, but leads to significantly reduced number and size of tumors with decreased cell proliferation, suggesting that the loss of Prx4 or Prx4/Srx renders mice partially resistant to urethane-induced lung tumorigenesis.

### 3.2. Depletion of Prx4 or Prx4/Srx Reduces Intratumoral Macrophage Infiltration

**Figure 2:**
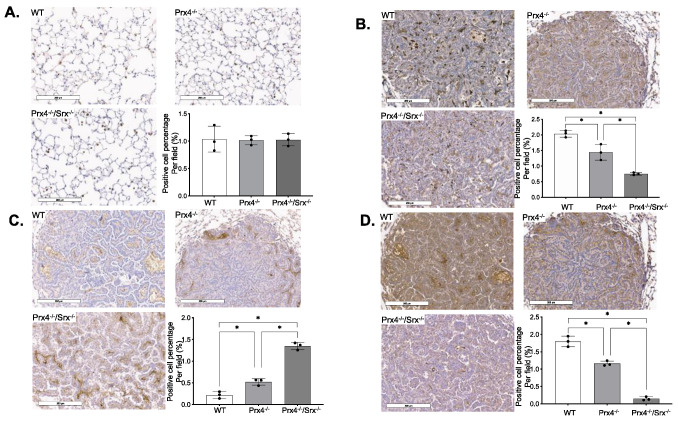
Prx4-Srx axis contributes to macrophage repolarization in the urethane-induced lung adenocarcinoma mouse model. (**A**) Anti-F4/80 staining of normal tissue from three genotypes and quantitative analysis of staining intensity. (**B**) Anti-F4/80 staining of tumor tissue from three genotypes and quantitative analysis of staining intensity. (**C**) Anti-CD86 staining of tumor tissue from three genotypes and quantitative analysis of staining intensity. (**D**) Anti-CD163 staining of tumor tissue from three genotypes and quantitative analysis of staining intensity. (*, *p* < 0.05).

The causal connection between ROS, inflammation, immunity, and cancer is increasingly acknowledged. Recent evidence has bolstered the notion that inflammation is a crucial aspect of tumor growth [44]. It is becoming increasingly apparent that the tumor microenvironment, which is predominantly regulated by inflammatory cells, is a crucial component of the neoplastic process, promoting proliferation, survival, and migration. Previous work has shown that mice treated with urethane have an elevated number of immune cells [45]. These findings suggest that increased lung inflammation is directly linked to the formation and progression of urethane-induced lung cancer. We wondered whether there was a difference in inflammatory cell infiltration among wild-type, Prx4^−/−^, and Prx4^−/−^/Srx^−/−^ mice tumors. F4/80 is a cell surface glycoprotein and has been widely used as a marker for mouse macrophages [46]. Firstly, we examined the staining of F4/80 positive cells in lungs of age-matched, naïve mice (without exposure to any treatment) from different genotypes. In general, there was an average of one F4/80^+^ cell with characteristic morphology of mature macrophage per alveoli, and there was no difference in staining intensity, pattern, or distribution (Figure 2A). However, F4/80 positive macrophages were abundantly found in urethane-induced lung tumors, and the overall number of macrophages in tumors from either Prx4^−/−^ or Prx4^−/−^/Srx^−/−^ mice was significantly less than in that of tumors from wild-type mice. Compared with tumors from Prx4^−/−^ mice, the number of F4/80^+^ cells in tumors from Prx4^−/−^/Srx^−/−^ mice was also significantly lower (Figure 2B). To further characterize these cells, staining for CD86 as a marker of M1 macrophages and CD163 as a marker of M2 macrophages were used. We found that macrophages in either Prx4^−/−^ or Srx^−/−^Prx4^−/−^ mice were mainly M1 type, whereas tumors from wild-type mice were enriched with M2 type (Figure 2C,D). These data indicate that the disruption of the Srx-Prx4 axis is associated with significant changes in intratumoral macrophage infiltration and their polarization, which may contribute to the reduction of tumor multiplicity and bulk observed in Prx4^−/−^ and Srx^−/−^Prx4^−/−^ mice. 

### 3.3. Exposure to Urethane Activates Srx and Prx4 Expression Which Contributes to Cell Transformation

**Figure 3:**
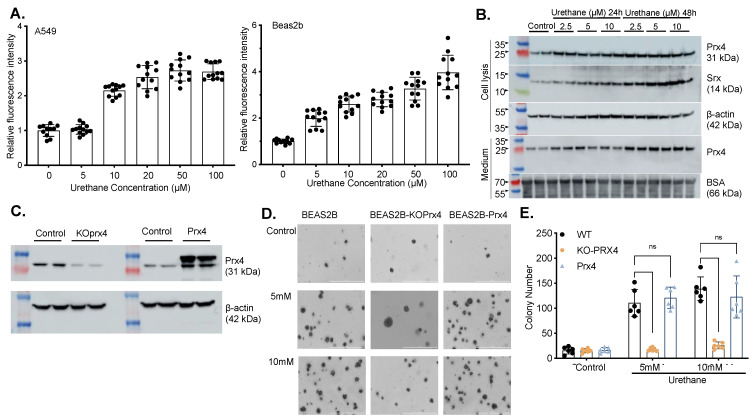
Urethane induces ROS level and cell transformation through increased Srx expression and Prx4 secretion in normal lung epithelial cells. (**A**) ROS levels change with urethane treatment in normal and cancer cell lines. (**B**) Change of intracellular Srx and Prx4 levels and secreted Prx4 with urethane treatment. (**C**) Confirmation of establishment of knockdown/overexpress Prx4 cell line (**D**,**E**) Change of cell transformation with urethane treatment. (ns, *p* ≥ 0.05).

To understand the mechanistic connection between urethane-induced lung tumorigenesis and the Srx-Prx4 axis, we asked whether exposure to urethane affects cellular redox homeostasis. Urethane (0–100 μM) was applied to both normal lung epithelial cells and cancer cells in culture to examine its effects on intracellular ROS levels using H2-DCFDA as the probe. We found that treatment of either BEAS2B or A549 cells leads to dose-dependent increased levels of ROS (Figure 3A). In our previous study, we showed that Srx promotes lung cancer progression by interacting with and activating the Prx family, including Prx1 and Prx4 [24]. Both cigarette smoke condensate and urethane induced the expression of Srx, Prx1, and Prx2 in human, normal bronchi epithelial cells [25]. Similarly, we found that exposure to urethane leads to a dose- and time-dependent activation of Srx and Prx4 in BEAS2B cells. In particular, urethane also stimulates the secretion of Prx4 into the culture medium (Figure 3B). Previously, we showed that urethane-induced Srx expression contributed to lung epithelial cell transformation; next we asked whether Prx4 is also involved in this process. Stable cell lines that were depleted of endogenous Prx4 expression or overexpression of a Flag-tagged Prx4 were established using BEAS2B cells (Figure 3C). Anchorage-independent colony formation in soft agar is a semi-quantitative assessment of cell transformation [47]. We found that urethane treatment of BEAS2B cells led to the dose-dependent increased number of colonies, whereas this phenotype was severely compromised in the absence of Prx4, although there was no further increase in cells that overexpress Prx4 (Figure 3D,E). These data indicate that Prx4 is also required for the urethane-induced transformation of lung epithelial cells.

### 3.4. Bioinformatics Analysis of Prx4

#### 3.4.1. Prx4 Is Significantly Upregulated in LUAD Patients and Is Negatively Correlated with Patient Survival and Prognosis 

**Figure 4:**
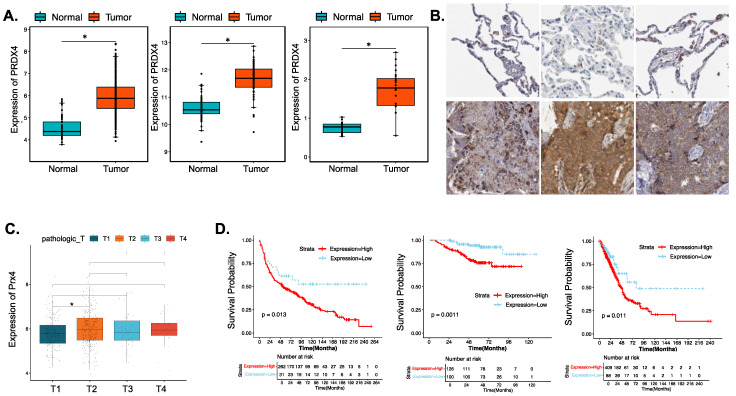
Prx4 is upregulated in lung adenocarcinoma, and negatively related to prognosis. (**A**) The expression level of PRDX4 in LUAD and normal tissues from TCGA-LUAD, GSE19804, and GSE27262 datasets. (**B**) Example results of anti-Prx4 staining of normal (upper) and tumor (lower) tissue from six patient samples obtained from Human Protein Atlas. (**C**)The expression level of Prx4 in different stages of LUAD. (**D**) Impact of PRDX4 expression on overall survival in LUAD patients. (*, *p* < 0.05).

To investigate the significance of Prx4 in human lung cancer, a meta-analysis of multiple existing datasets was performed. We found that Px4 was significantly upregulated in lung cancer compared with normal lungs. Figure 4A shows example results from TCGA-LUAD, GSE19804, and GSE27262 datasets. Immunohistochemical results of Prx4 in LUAD tissue and normal tissue from Human Protein Atlas (image available from: v22/proteinatlas.org) [35] showed a higher level of Prx4 protein expression in LUAD, which is consistent with the gene expression level (Figure 4B). Box plot results for Prx4 with different clinical features showed that Prx4 expression basically tended to increase as pathologic T-staging progressed, with significant levels between T1 and T2 staging (Figure 4C). Using TCGA-LUAD, the K-M analysis showed that patients with high levels of Prx4 expression had a significantly lower probability of OS (*p* = 0.011). Additionally, there were differences in OS between Prx4 high/low groups in the GSE31210 and GSE30219 datasets (Figure 4D). From the perspective of clinical correlation of the Srx-Prx4 axis in LUAD, the expression heatmap of SRXN1 and PRDX4 in LUAD patients with different clinical features is demonstrated in Figure S3A (Supplementary Materials), and the significances of different clinical features between the high- and low-expression groups of SRXN1 were calculated using chi-square test and are exhibited in Table S1, indicating that SRXN1 expression might be correlated to pathologic M (*p* = 0.002377), tobacco smoking history (*p* = 0.02913), gender (*p* = 3.342 × 10^−5^), as well as tumor stage (*p* = 0.003266). Furthermore, Wilcoxon test and analysis of variance (ANOVA) were performed for the expression investigation of SRXN1 in different groups with clinical features which were associated with SRXN1 expression. As shown in Figure S3B, SRXN1 was more highly expressed in male cases (*p* = 4.4 × 10^−4^) and the LUAD patients with a tobacco-smoking history (*p* = 3.6 × 10^−3^); meanwhile, SRXN1 expression gradually increased as tumor stage progressed (*p* = 0.01). The significance of different clinical features in the Prx4 high/low groups was shown in Table 1. Among cases in the Prx4 low expression group, a third of patients were <65 years old and 2/3 cases were >65 years old. In the Prx4 high expression group, patients < 65 years old and ≥65 years old each accounted for approximately half of the patients, which indicated that younger lung cancer patients were more likely to be found in the Prx4 high group. The expression results of SRXN1 revealed the overexpression of SRXN1 in the LUAD groups of both TCGA and GSE27262 datasets (*p* < 0.05) (Figure S1A). Next, the Kaplan-Meier (K-M) survival analysis confirmed that SRXN1 expression was inversely associated with OS rate of LUAD patients (Figure S1B).

#### 3.4.2. Prx4 May Affect the Tumor Microenvironment of LUAD

**Figure 5:**
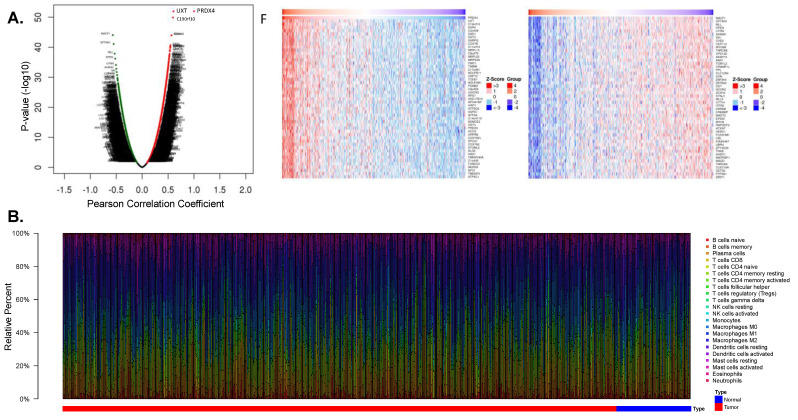

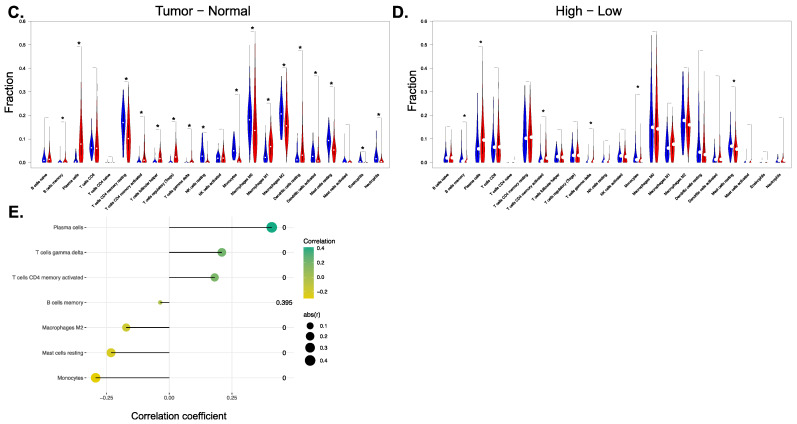
PRDX4 regulates tumor immune microenvironment in LUAD. (**A**) Gene association with PRDX4 in LUAD. (**B**,**C**) The 22 infiltrated immune cells in LUAD and normal tissues. (**D**) The 22 infiltrated immune cells in the PRX4 high/low expression LUAD groups. (**E**) Correlation between PRDX4 and the seven differential immune cells, (*, *p* < 0.05).

The volcano map and heat map of genes with significant positive and negative associations with PRDX4 are shown in Figure 5A. The results showed that the gene most positively associated with PRX4 was UXT and the gene most negatively associated with PRX4 was MACF1. GSEA functional enrichment analysis showed that Prx4 was enriched to rap1 signaling, Th17 cell differentiation, hippo signaling, JAK-STAT signaling, MAPK signaling, Th1 and Th2 cell differentiation, ErbB signaling, and other key pathways. In order to further explore the potential biological alterations relevant to SRXN1 expression, the differentially expressed genes (DEGs) between the high- and low-expression groups of SRXN1 were screened and displayed in Figure S2A,B, where 81 out of 139 DEGs were upregulated in tumor groups (adj. *p* Value < 0.05 and |log2fold change| > 1). Additionally, GO and KEGG analyses were conducted to interpret the biological significance of these DEGs, and identified the KEGG pathway of glutathione metabolism and the GO terms of response to toxic substances and oxidoreductase activity, acting on the CH-OH group of donors (Figure S2C,D).

Tumor-infiltrating immune cells (TIICs) can be targeted for chemo-radiation therapy [48]. The abundance of each immune cell in each sample was calculated by the CIBERSORT algorithm, and the samples with *p* > 0.05 were excluded, leaving 472 LUAD samples and 57 normal samples. The scoring heat map of 22 immune cells is shown in Figure 5B. There were 18 immune cells with significant differences between LUAD and normal samples (Figure 5C): monocytes, macrophages M2, plasma cells, B cells memory, T-cells CD4 memory activated, T-cells gamma delta, mast cells resting, macrophages M0, NK cells activated, T-cells CD4 memory resting, neutrophils, macrophages M1, T-cells follicular helper, eosinophils, dendritic cells resting, NK cells resting, dendritic cells activated, and T-cells regulatory. The violin plot of the abundance of the 22 immune cells in the PRDX4 high/low expression groups is shown in Figure 5D, which shows that seven immune cells had significant differences: monocytes, macrophages M2, plasma cells, B cells memory, T-cells CD4 memory activated, T-cells gamma delta, and mast cells resting. A total of seven common differential immune cells were obtained by taking the intersection of the above two groups of differential immune cells. The correlation between PRDX4 and the seven differential immune cells is shown in Figure 5E. According to the correlation coefficient |r| > 0.3, *p* < 0.05, there was a significant positive correlation between PRDX4 and plasma cells (Table 2).

#### 3.4.3. Investigation of the Biological Mechanism of Abnormal Expression of PRX4

**Figure 6:**
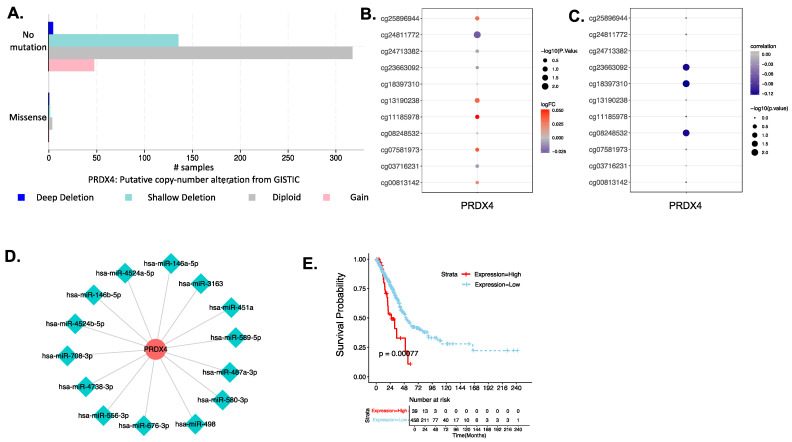
Prediction and investigation of the biological mechanism of Prx4 in LUAD. (**A**) The genetic alteration of PRDX4 in LUAD from cBioPortal. (**B**) Methylation differences between LUAD and normal tissues. (**C**) Spearman correlation coefficient of methylation and gene expression. (**D**) miRNA prediction from miRwalk and ENCORI database. (**E**) Impact of has-miR-3163 on overall survival of LUAD patients.

The genetic mutation frequency and mutation characteristics of PRDX4 in LUAD analyzed by cBioPortal are shown in Figure 6A. The differential analysis between LUAD and normal samples obtained one differentially downregulated methylation site cg24811772 (Figure 6B). Additionally, the correlation bubble chart between Prx4 expression level and its corresponding methylation sites is shown in Figure 6C. Prx4 had a weak negative correlation with cg08248532, cg18397310 and cg23663092. The miRwalk database predicted 2 miRNAs (hsa-miR-708-3P; hsa-miR-4738-3P), while the ENCORI database predicted 12 miRNAs (Table 3). The combined results of the two databases yielded a total of 14 miRNA-mRNA regulatory relationship pairs, and the miRNA- mRNA regulatory network is shown in Figure 6D. The miRNA expression data of LUAD cases in the TCGA database were matched with the above 14 miRNAs, and a total of 12 miRNA expression data were obtained. Univariate cox regression analysis yielded 1 miRNA: hsa-miR-3163 (Table 4). The 497 patients were divided into hsa-miR-3163 high expression group (39 patients) and hsa-miR-3163 low expression group (458 patients) according to the mean of hsa-miR-3163 expression. The results of K-M curve analysis showed that hsa-miR-3163 high/low expression groups were significantly correlated with OS (Figure 6E), thus hsa-miR-3163 was predicted as a key miRNA in the post-transcriptional regulation of PRX4 affecting prognosis of LUAD.

## 4. Discussion

Compared to most other tissues, the lung is directly exposed to higher oxygen concentrations. Smoking, inflammation, pollution, toxins, and carcinogens all raise the number of reactive oxygen species (ROS) in the lungs, which then damage DNA through a free radical-related mechanism. It is clear that the imbalance between the pulmonary defense systems and cellular redox state affects both the pathophysiology and development of malignant lung illnesses. Asthma and chronic obstructive pulmonary disease are examples of non-malignant lung illnesses where lung antioxidant levels are known to be lower [49], but lung cancers are known to have high levels of primary antioxidant mechanisms. Cancerous cells are abnormal, uncontrollably reproducing cells. Although transient intracellular ROS production is required for cell signaling, persistent production is damaging to all cells, including cancer cells. Free radicals are firmly connected to both carcinogenesis and tumor behavior. The altered pro-oxidant intracellular environment that inactivates tumor suppression genes, and/or activates oncogenes with subsequent changes in cell growth, survival, and apoptosis is an accepted major theory that explains the significance of oxidants and imbalance of the cellular redox state in lung carcinogenesis [50]. Thus, cancer cells need increased intracellular ROS levels for continuous proliferation. However, in order to maintain ROS homeostasis, cancer cells overexpress antioxidant enzymes such as Prx4 and Srx. It has been demonstrated that the tumor cells’ high antioxidant capacity plays a crucial role in cancer biology, with cellular redox status serving as one of the key regulators of several growth factors linked to angiogenesis and cell proliferation.

The main cellular antioxidant axis with peroxidase activity is the Prx4-Srx. These two antioxidants have both been found to be overexpressed in a variety of tumor forms, including lung cancer. It is unclear how these antioxidants affect the development of lung cancer brought on by cigarette smoke or chemical toxins. Under normal circumstances, they facilitate redox signaling by forming intramolecular disulfide bonds, whereas under oxidative stress, they scavenge hydrogen peroxide by peroxidation [51]. A variety of intracellular signal pathways are transduced by Prx4 activity, and these pathways are essential for both the physiological and pathological functions in mammalian cells. Srx is a special enzyme that lowers hyperoxidized Prx4. Although we previously showed that the Srx is essential for the invasion and metastasis of lung cancer cells, the functional importance of this antioxidant axis in lung tumorigenesis was still unclear and needed to be explored with further research. In this study, we investigated the distinct signaling functions of the Prx4-Srx axis in relation to cancer cell proliferation and prognosis, in addition to their intrinsic ROS—scavenging activity.

Previously, we determined that both Prx4 and Srx reduced anchorage-independent colony formation, cell migration, and invasion in vitro [24]; subsequently, we found the contribution of Srx in smoking-related lung tumorigenesis in vivo [25]. Now we provide solid evidence that the Prx4-Srx axis is not only required for urethane-induced tumor formation and progression in vivo, but can also regulate tumor-associated macrophages (TAM) through repolarizing intratumoral macrophages.

The urethane-induced lung cancer mouse model is the most commonly used non-hereditary rodent model to simulate the development of LUAD in human patients. It is generally accepted that there are gender differences in mouse susceptibility to urethane, and tumor cell proliferation was found to be significantly lower in female animals [52]. The previously published study eliminated potential gender differences that could complicate the interpretation of results by utilizing only female Srx^−/−^ mice [25]. In the current study, we used both female and male mice and demonstrated that although male mice developed more urethane-induced tumors than females, Prx4^−/−^ and Prx4^−/−^/Srx^−/−^ mice have significantly lower tumor multiplicity and volume in both genders, which suggests that Prx4-Srx depletion confers resistance to urethane-induced lung cancer. Compared to those of WT mice, tumors from Prx4-Srx deficient mice exhibited significantly decreased rates of cell proliferation. Although normal lung tissue showed a low expression level of Prx4 and Srx, both antioxidants were highly expressed in urethane-induced tumors in WT mice. Based on the primary function of Prx4-Srx, increased expression of the axis in tumors may help the tumor cells increase their resistance to oxidative stress through scavenging H_2_O_2_. Thus, tumors derived from Prx4^−/−^ and Prx4^−/−^/Srx^−/−^ mice are likely to be more sensitive to oxidative stress, which may combine with the lower cell proliferation rates to partially explain the decreased tumor multiplicity and volume found in Prx4^−/−^ and Prx4^−/−^/Srx^−/−^ mice. Our bioinformatics analysis is consistent with the in vivo results. We identified that both SRXN1 and PRDX4 genes are upregulated in LUAD tumor tissue and correlated with the cancer development. LUAD cancer patients with higher Srx-Prx4 expression show a poor prognosis. Importantly, GO and KEGG analysis revealed that Prx4 was enriched to several critical pathways which are widely recognized to be included in cancer progression, including rap1 signaling, hippo signaling, JAK-STAT signaling, MAPK signaling, ErbB signaling, and other key pathways. Actually, Dr. Wei demonstrated that the Srx-Prx4 axis is required for sufficient activation of MAPK pathway, and suggested that Srx-Prx4 promoted tumor progression through the regulation of the MAPK pathway when our lab first identified this antioxidant axis and its critical role in human cancer maintenance and metastasis [24]. We will explore the other enriched pathway in a further study.

In our attempts to comprehend the regulation of the immune environment in urethane-induced lung cancer, we demonstrate that macrophages enriched in tumor tissue and loss of Prx4 or Prx4/Srx cause an apparent intratumoral TAM reduction and repolarization of macrophages from the M2 to M1 phase. The tumor microenvironment is a complex and dynamic ecosystem that supports tumor cells as they progress through malignancy. Macrophages are one of the most abundant immune cells among the innate and adaptive immune cells recruited to the tumor site [53]. Despite being somewhat contentious, clinical studies and experimental mouse models suggest that TAM plays a pro-tumoral role in general [54]. Both M1- and M2-polarized macrophages have been identified in tumor microenvironments. In particular, M2 subtypes aided lung cancer invasion and tumor growth, while the M1 subtype inhibited angiogenesis, induced apoptosis, and increased sensitivity to the chemotherapy [55]. In the present bioinformatics study, we discovered that the ratio of M1/M2 was significantly correlated with the extended OS for LUAD patients, implying that the rate of M1/M2 could be a target of immune therapy research for lung cancer patients. However, it is still unknown how the imbalance of ROS and antioxidants affect the TAM and macrophage polarization in the cancer microenvironment, as well as the regulation of tumor progression and prognosis. It has been shown that the Prx4-Srx axis exists in the macrophages regulated by Nrf2 [56,57], and that the expression of Prx4 elevates inflammatory cytokines including interleukin-1 beta (IL-1*β*) and matrix metallopeptidase 9 (MMP9) [22]. The expression of Prx4-Srx may increase the antioxidant capacity of macrophages, which may also be involved in the LUAD patients’ smoking-related immune response. Then, in our study, depletion of Prx4-Srx disrupted communication between the tumor and its microenvironment and regulated macrophage enrichment and polarization by modifying secreted cytokine and chemokine. However, we still do not know the mechanism(s) of the macrophage alteration for infiltration and polarization regulated by Prx4-Srx. Meanwhile, in adaptive immunity, M1- and M2-type macrophages inevitably direct T cells aimed at Th1- or Th2-like activities, and these macrophage conduct activities are the primary directing factor in immune responses. The Kyoto Encyclopedia indicated that Prx4 was enriched to Th1, Th2, and Th17 differentiation. Thus, the reduction in total macrophage infiltrate and increase in M1/M2 with urethane treatment may contribute to the decrease in lung tumor multiplicity and volume we observed in both Prx4^−/−^ and Prx4^−/−^/Srx^−/−^ mice. There are clear imbalances in M1/M2 in many modern diseases, such as cancer. Correction of this imbalance may lead to improved health [58]. Thus, the mechanism of the repolarization of macrophages and whether there are differences in other immune cells, such as Th1- and Th2-type cells, regulated by macrophages and the Prx4-Srx axis need to be answered in future research.

To transfer these results from the bench to clinics, from mice to patients, this study revealed that Srx-Prx4 axis is upregulated and genitively regulates the prognosis of LUAD patients. According to our results on the role of this antioxidant axis in LUAD, some patients with LUAD may benefit from Srx-Prx4 interruption. A specific Srx inhibitor, K27, has been identified by Kim et al. in 2016 and showed a cancer-cell killing effect through the accumulation of ROS and mitochondrial dysfunction [59]. Furthermore, they also indicated that inhibition of Srx may result in a decrease in reduction in 2-cys Prxs, leading to oxidative damage and cell apoptosis. Some general Prx inhibitors, such as Frenolicin B [60] and ConoidinA [61], are being investigated as potential cancer treatments. However, specific inhibitors targeting Prx4 have not yet been found despite being a very promising therapeutic target for the prevention and treatment of cancer. Additionally, cancer cells’ resistance to chemo/radiotherapy is frequently attributed to the increase in antioxidant capacity as an adaptive response to oxidative stress. To increase the therapeutic efficacy of conventional cancer treatments, using Srx-Prx4 inhibitors in combination with them may be a potential approach by decreasing cancer cell resistance. Our lab demonstrated that a reduced Prx4 level elevated cancer cell sensitivity to radioresistance in prostate cancer in vitro and in vivo [62]. The discovery of certain Prx4 inhibitors in the future would advance this area.

Currently, there is a great interest in identifying clinically relevant biomarkers. Research on Prx4 has uncovered that Prx4 levels in the blood of patients with acute or chronic diseases differ from healthy individuals [63]. The most striking research findings are the feasibility and clinical significance of Prx4 as a multifunctional staging and prognosis biomarker. Based on our findings that urethane induces tumorigenesis [25] and significantly stimulates Prx4 secretion in normal lung epithelial cells, we make the bold assumption that extracellular Prx4 levels may not only reflect the oxidative state, but also the tumor status of patients. Actually, Prx4 in the circulation system has been revealed the pro-metastasis function through mediating osteoclast activation in breast [64] and prostate cancer [65]. Additionally, the potential prognostic and diagnostic role of secreted Prx4 were also been recognized in these two types of cancer [66]. In light of the LUAD current biomarker situation, it will be valuable to establish a proper model and explore the potential of Prx4 as a high-utility, non-invasive tool for early diagnosis and dependable patient monitoring in lung cancer patients.

In summary, this study demonstrates that disruption of the Prx4-Srx axis leads to a corresponding reduction of tumor growth in the urethane-induced mouse model, revealing a unique oncogenic function of Prx4-Srx in human lung adenocarcinoma. Srx-Prx4 is a potential target of combination with chemotherapy and radiotherapy for LUAD patients. Secreted Prx4 in patients’ blood can serve as a biomarker for diagnosis and treatment response prediction. The insights into macrophage repolarization provide a dramatic change in the understanding of how immune systems operate and foster new anti-inflammatory therapeutic approaches to cancer development. Most importantly, this discovery is illuminating entirely new strategies for immunotherapy in LUAD. Given the current study’s resource limitations, we were unable to look into whether and how cellular signaling modification caused by Prx4 depletion contributes to LUAD metastasis and chemo/radio-resistance. Moreover, the mechanism of the regulation of the tumor immune environment in Srx-Prx4 depletion mice needs to be further studied.

## 5. Conclusions

The upregulated Prx4-Srx axis promotes LUAD development and is negatively correlated with prognosis by interrupting ROS/antioxidant equilibrium and regulating the immune environment. This study offers some novel perspectives on the antioxidant therapy for cancer. Further studies will focus on investigating the mechanism(s) of the microenvironment alteration and exploring the benefit of intervention on this axis in cancer treatment.

## Figures and Tables

**Table 1:** Clinical characteristics of LUAD patients between high- and low-expression groups of PRDX4.

Features	H_Prx4 (*n* = 409)	L_PRDX4 (*n* = 88)	*p* Value
age			0.01047
age < 65	190	28	
age ≥ 65	213	56	
pathologic_M			0.3475
M0	278	53	
M1	19	5	
pathologic_N			0.473
N0	259	62	
N1	82	12	
N2	58	11	
N3	2	0	
pathologic_T			0.01243
T1	131	35	
T2	227	40	
T3	36	7	
T4	15	3	
tobacco_smoking_history			0.7146
NO	56	15	
YES	341	71	
gender			0.7899
female	223	46	
male	186	42	
tissue_or_origin			0.6715
Lower lobe	137	31	
Middle lobe	17	4	
Upper lobe	239	52	
tumor_stage			0.6122
stage i	216	51	
stage ii	102	16	
stage iii	66	14	
stage iv	19	6	

**Table 2:** The correlation between PRDX4 and the seven differential immune cells.

Cell	Gene	r	*p* Value	Q Value
B cells memory	Prx4	−0.0357658	0.39488518	0.39488518
Plasma cells	Prx4	0.40876086	2.75 × 10^−24^	1.93 × 10^−23^
T cells CD4 memory activated	Prx4	0.18108563	1.41 × 10^−5^	1.97 × 10^−5^
T cells gamma delta	Prx4	0.20982468	4.51 × 10^−7^	7.90 × 10^−7^
Monocytes	Prx4	−0.2933115	9.86 × 10^−13^	3.45 × 10^−12^
Macrophages M2	Prx4	−0.1713956	4.13 × 10^−5^	4.81 × 10^−5^
Mast cells resting	Prx4	−0.2319601	2.24 × 10^−8^	5.22 × 10^−8^

**Table 3:** The 12 predicted miRNA from ENCORI database.

miRNA ID	miRNA Name	geneID	Gene Name	Gene Type	Chromosome	Narrow Start	Narrow End	Broad Start	Broad End	Strand	clipExp Num	DegraExp Num	PITA	RNA22	miRmap	microT	miRanda	Pancancer Num
MIMAT0000449	hsa-miR-146a-5p	ENSG00000123131	Prx4	protein_coding	chrX	23704483	23704489	23704469	23704490	+	5	0	1	0	1	0	1	6
MIMAT0001631	hsa-miR-451a	ENSG00000123131	Prx4	protein_coding	chrX	23704500	23704505	23704485	23704506	+	3	0	1	0	0	0	1	1
MIMAT0002178	hsa-miR-487a-3p	ENSG00000123131	Prx4	protein_coding	chrX	23704470	23704475	23704470	23704475	+	5	0	1	0	0	0	0	6
MIMAT0002809	hsa-miR-146b-5p	ENSG00000123131	Prx4	protein_coding	chrX	23704483	23704489	23704466	23704490	+	5	0	1	0	1	0	1	3
MIMAT0002824	hsa-miR-498	ENSG00000123131	Prx4	protein_coding	chrX	23704476	23704482	23704476	23704482	+	4	0	1	0	1	0	0	4
MIMAT0003245	hsa-miR-580-3p	ENSG00000123131	Prx4	protein_coding	chrX	23704485	23704490	23704485	23704490	+	3	0	1	0	0	0	0	2
MIMAT0004793	hsa-miR-556-3p	ENSG00000123131	Prx4	protein_coding	chrX	23693402	23693407	23693402	23693407	+	1	0	0	0	1	0	0	3
MIMAT0004799	hsa-miR-589-5p	ENSG00000123131	Prx4	protein_coding	chrX	23704484	23704489	23704484	23704489	+	3	0	1	0	0	0	0	3
MIMAT0015037	hsa-miR-3163	ENSG00000123131	Prx4	protein_coding	chrX	23693418	23693446	23693418	23693446	+	1	0	0	0	0	1	0	2
MIMAT0018204	hsa-miR-676-3p	ENSG00000123131	Prx4	protein_coding	chrX	23693424	23693452	23693424	23693452	+	1	0	0	0	0	1	0	3
MIMAT0019062	hsa-miR-4524a-5p	ENSG00000123131	Prx4	protein_coding	chrX	23693414	23693420	23693393	23693421	+	1	0	0	0	1	1	0	1
MIMAT0022255	hsa-miR-4524b-5p	ENSG00000123131	Prx4	protein_coding	chrX	23693414	23693420	23693393	23693421	+	1	0	0	0	1	1	0	0

**Table 4:** Cos regression analysis of the 12 predicted miRNA from the ENCORI database.

HR	Lower.95	Upper.95
hsa-miR-146a-5p	0.91033546	0.79799012
hsa-miR-146b-5p	0.94493836	0.81559882
hsa-miR-3163	12.3304215	2.93772613
hsa-miR-451a	0.98124854	0.8963051
hsa-miR-4524a-5p	0.84779499	0.35083517
hsa-miR-4738-3p	0.85010695	0.53104859
hsa-miR-487a-3p	1.10794343	0.95751824
hsa-miR-556-3p	0.86824518	0.68867783
hsa-miR-580-3p	0.86960466	0.65553948
hsa-miR-589-5p	1.04741951	0.86650936
hsa-miR-676-3p	0.9633758	0.78058721
hsa-miR-708-3p	0.98595142	0.86659775

## Data Availability

Not applicable.

## Supplementary Materials

The following are available online at https://www.mdpi.com/article/10.3390/antiox12020367/s1, Figure S1: SRXN1 is up-regulated in lung adenocarcinoma, and negatively related to prognosis., Figure S2: Identification and exploration of DEGs between the high- and low-expression groups of SRXN1, Figure S3: The correlation analysis of SRXN1 expression and different clinical features. Table S1: Clinical characteristic of LUAD patients between the high and low expression groups of SRXN1.

## Abbreviations

Srx (Sulfiredoxin); Prxs (Peroxiredoxins); Prx4 (Peroxiredoxin 4); LUAD (lung adenocarcinoma); KO (Peroxiredoxin 4-knockout); DKO (Peroxiredoxin 4/Sulfiredoxin double-knockout).

## References

[1] Siegel R.L., Miller K.D., Fuchs H.E., Jemal A. (2022). Cancer statistics, 2022. Cancer J. Clin..

[2] Islami F., Goding Sauer A., Miller K.D., Siegel R.L., Fedewa S.A., Jacobs E.J., McCullough M.L., Patel A.V., Ma J., Soerjomataram I. (2018). Proportion and number of cancer cases and deaths attributable to potentially modifiable risk factors in the United States. Cancer J. Clin..

[3] Giotopoulou G.A., Stathopoulos G.T. (2020). Effects of Inhaled Tobacco Smoke on the Pulmonary Tumor Microenvironment. Tumor Microenviron..

[4] Saikolappan S., Kumar B., Shishodia G., Koul S., Koul H.K. (2019). Reactive oxygen species and cancer: A complex interaction. Cancer Lett..

[5] Goldkorn T., Filosto S., Chung S. (2014). Lung injury and lung cancer caused by cigarette smoke-induced oxidative stress: Molecular mechanisms and therapeutic opportunities involving the ceramide-generating machinery and epidermal growth factor receptor. Antioxid. Redox Signal..

[6] Hayes J.D., Dinkova-Kostova A.T., Tew K.D. (2020). Oxidative stress in cancer. Cancer Cell.

[7] Reuter S., Gupta S.C., Chaturvedi M.M., Aggarwal B.B. (2010). Oxidative stress, inflammation, and cancer: How are they linked?. Free Radic. Biol. Med..

[8] Chae H.Z., Robison K., Poole L.B., Church G., Storz G., Rhee S.G. (1994). Cloning and sequencing of thiol-specific antioxidant from mammalian brain: Alkyl hydroperoxide reductase and thiol-specific antioxidant define a large family of antioxidant enzymes. Proc. Natl. Acad. Sci. USA.

[9] Seo M.S., Kang S.W., Kim K., Baines I.C., Lee T.H., Rhee S.G. (2000). Identification of a new type of mammalian peroxiredoxin that forms an intramolecular disulfide as a reaction intermediate. J. Biol. Chem..

[10] Chua P.-J., Lee E.-H., Yu Y., Yip G.W.-C., Tan P.-H., Bay B.-H. (2010). Silencing the Peroxiredoxin III gene inhibits cell proliferation in breast cancer. Int. J. Oncol..

[11] Zhang Y., Sun C., Xiao G., Shan H., Tang L., Yi Y., Yu W., Gu Y. (2019). S-nitrosylation of the Peroxiredoxin-2 promotes S-nitrosoglutathione-mediated lung cancer cells apoptosis via AMPK-SIRT1 pathway. Cell Death Dis..

[12] Chang X.-Z., Li D.-Q., Hou Y.-F., Wu J., Lu J.-S., Di G.-H., Jin W., Ou Z.-L., Shen Z.-Z., Shao Z.-M. (2007). Identification of the functional role of peroxiredoxin 6 in the progression of breast cancer. Breast Cancer Res..

[13] Wang T., Tamae D., LeBon T., Shively J.E., Yen Y., Li J.J. (2005). The role of peroxiredoxin II in radiation-resistant MCF-7 breast cancer cells. Cancer Res..

[14] Okado-Matsumoto A., Matsumoto A., Fujii J., Taniguchi N. (2000). Peroxiredoxin IV is a secretable protein with heparin-binding properties under reduced conditions. J. Biochem..

[15] Fujii J., Ikeda Y. (2002). Advances in our understanding of peroxiredoxin, a multifunctional, mammalian redox protein. Redox Rep..

[16] Schulte J., Struck J., Köhrle J., Müller B. (2011). Circulating levels of peroxiredoxin 4 as a novel biomarker of oxidative stress in patients with sepsis. Shock.

[17] Gerrits E.G., Alkhalaf A., Landman G.W., van Hateren K.J., Groenier K.H., Struck J., Schulte J., Gans R.O., Bakker S.J., Kleefstra N. (2014). Serum peroxiredoxin 4: A marker of oxidative stress associated with mortality in type 2 diabetes (ZODIAC-28). PLoS ONE.

[18] Nawata A., Noguchi H., Mazaki Y., Kurahashi T., Izumi H., Wang K.-Y., Guo X., Uramoto H., Kohno K., Taniguchi H. (2016). Overexpression of peroxiredoxin 4 affects intestinal function in a dietary mouse model of nonalcoholic fatty liver disease. PLoS ONE.

[19] Kam M.K., Lee D.G., Kim B., Lee H.-S., Lee S.-R., Bae Y.C., Lee D.-S. (2019). Peroxiredoxin 4 ameliorates amyloid beta oligomer-mediated apoptosis by inhibiting ER-stress in HT-22 hippocampal neuron cells. Cell Biol. Toxicol..

[20] Guo X., Yamada S., Tanimoto A., Ding Y., Wang K.-Y., Shimajiri S., Murata Y., Kimura S., Tasaki T., Nabeshima A. (2012). Overexpression of peroxiredoxin 4 attenuates atherosclerosis in apolipoprotein E knockout mice. Antioxid. Redox Signal..

[21] Basu A., Banerjee H., Rojas H., Martinez S.R., Roy S., Jia Z., Lilly M.B., De León M., Casiano C.A. (2011). Differential expression of peroxiredoxins in prostate cancer: Consistent upregulation of PRDX3 and PRDX4. Prostate.

[22] Zheng J., Guo X., Nakamura Y., Zhou X., Yamaguchi R., Zhang J., Ishigaki Y., Uramoto H., Yamada S. (2020). Overexpression of PRDX4 Modulates Tumor Microenvironment and Promotes Urethane-Induced Lung Tumorigenesis. Oxidative Med. Cell. Longev..

[23] Gregorieff A., Clevers H. (2005). Wnt signaling in the intestinal epithelium: From endoderm to cancer. Genes Dev..

[24] Wei Q., Jiang H., Xiao Z., Baker A., Young M.R., Veenstra T.D., Colburn N.H. (2011). Sulfiredoxin–peroxiredoxin IV axis promotes human lung cancer progression through modulation of specific phosphokinase signaling. Proc. Natl. Acad. Sci. USA.

[25] Mishra M., Jiang H., Chawsheen H.A., Gerard M., Toledano M.B., Wei Q. (2018). Nrf2-activated expression of sulfiredoxin contributes to urethane-induced lung tumorigenesis. Cancer Lett..

[26] Kang S.W., Baines I.C., Rhee S.G. (1998). Characterization of a mammalian peroxiredoxin that contains one conserved cysteine. J. Biol. Chem..

[27] Nettleship A., Henshaw P.S., Meyer H.L. (1943). Induction of pulmonary tumors in mice with ethyl carbamate (urethane). J. Natl. Cancer Inst..

[28] Tuveson D.A., Jacks T. (1999). Modeling human lung cancer in mice: Similarities and shortcomings. Oncogene.

[29] Hanna J.M., Onaitis M.W. (2013). Cell of origin of lung cancer. J. Carcinog..

[30] Malkinson A.M. (1998). Molecular comparison of human and mouse pulmonary adenocarcinomas. Exp. Lung Res..

[31] Iuchi Y., Okada F., Tsunoda S., Kibe N., Shirasawa N., Ikawa M., Okabe M., Ikeda Y., Fujii J. (2009). Peroxiredoxin 4 knockout results in elevated spermatogenic cell death via oxidative stress. Biochem. J..

[32] Planson A.-G., Palais G., Abbas K., Gerard M., Couvelard L., Delaunay A., Baulande S., Drapier J.-C., Toledano M.B. (2011). Sulfiredoxin protects mice from lipopolysaccharide-induced endotoxic shock. Antioxid. Redox Signal..

[33] Jiang H., Wu L., Mishra M., Chawsheen H.A., Wei Q. (2014). Expression of peroxiredoxin 1 and 4 promotes human lung cancer malignancy. Am. J. Cancer Res..

[34] Eruslanov E., Kusmartsev S. (2010). Identification of ROS using oxidized DCFDA and flow-cytometry. Advanced Protocols in Oxidative Stress II.

[35] Uhlén M., Fagerberg L., Hallström B.M., Lindskog C., Oksvold P., Mardinoglu A., Sivertsson Å., Kampf C., Sjöstedt E., Asplund A. (2015). Tissue-based map of the human proteome. Science.

[36] Ozga A.J., Chow M.T., Luster A.D. (2021). Chemokines and the immune response to cancer. Immunity.

[37] Hänzelmann S., Castelo R., Guinney J. (2013). GSVA: Gene set variation analysis for microarray and RNA-seq data. BMC Bioinform..

[38] Aran D., Hu Z., Butte A.J. (2017). xCell: Digitally portraying the tissue cellular heterogeneity landscape. Genome Biol..

[39] Sturm G., Finotello F., List M. (2020). Immunedeconv: An R package for unified access to computational methods for estimating immune cell fractions from bulk RNA-sequencing data. Bioinformatics for Cancer Immunotherapy.

[40] Cerami E., Gao J., Dogrusoz U., Gross B.E., Sumer S.O., Aksoy B.A., Jacobsen A., Byrne C.J., Heuer M.L., Larsson E. (2012). The cBio cancer genomics portal: An open platform for exploring multidimensional cancer genomics data. Cancer Discov..

[41] Gao J., Aksoy B.A., Dogrusoz U., Dresdner G., Gross B., Sumer S.O., Sun Y., Jacobsen A., Sinha R., Larsson E. (2013). Integrative analysis of complex cancer genomics and clinical profiles using the cBioPortal. Sci. Signal..

[42] Ritchie M.E., Phipson B., Wu D., Hu Y., Law C.W., Shi W., Smyth G.K. (2015). limma powers differential expression analyses for RNA-sequencing and microarray studies. Nucleic Acids Res..

[43] Festing M.F., Yang A., Malkinson A. (1994). At least four genes and sex are associated with susceptibility to urethane-induced pulmonary adenomas in mice. Genet. Res..

[44] Coussens L.M., Werb Z. (2002). Inflammation and cancer. Nature.

[45] Xu C., Zhou L., Lu L., Chen T., Wei S., Lin X., Lian X. (2016). Inflammation has a role in urethane-induced lung cancer in C57BL/6J mice. Mol. Med. Rep..

[46] dos Anjos Cassado A. (2017). F4/80 as a major macrophage marker: The case of the peritoneum and spleen. Macrophages.

[47] De Larco J.E., Todaro G.J. (1978). Growth factors from murine sarcoma virus-transformed cells. Proc. Natl. Acad. Sci. USA.

[48] Ye L., Zhang T., Kang Z., Guo G., Sun Y., Lin K., Huang Q., Shi X., Ni Z., Ding N. (2019). Tumor-infiltrating immune cells act as a marker for prognosis in colorectal cancer. Front. Immunol..

[49] Kinnula V.L., Crapo J.D. (2003). Superoxide dismutases in the lung and human lung diseases. Am. J. Respir. Crit. Care Med..

[50] Burdon R.H. (1995). Superoxide and hydrogen peroxide in relation to mammalian cell proliferation. Free Radic. Biol. Med..

[51] Rhee S.G., Woo H.A., Kil I.S., Bae S.H. (2012). Peroxiredoxin functions as a peroxidase and a regulator and sensor of local peroxides. J. Biol. Chem..

[52] Stakišaitis D., Mozūraitė R., Kavaliauskaitė D., Šlekienė L., Balnytė I., Juodžiukynienė N., Valančiūtė A. (2017). Sex-related differences of urethane and sodium valproate effects on Ki-67 expression in urethane-induced lung tumors of mice. Exp. Ther. Med..

[53] Noy R., Pollard J.W. (2014). Tumor-associated macrophages: From mechanisms to therapy. Immunity.

[54] Biswas S.K., Allavena P., Mantovani A. (2013). Tumor-associated macrophages: Functional diversity, clinical significance, and open questions. Semin. Immunopathol..

[55] Yuan A., Hsiao Y.-J., Chen H.-Y., Chen H.-W., Ho C.-C., Chen Y.-Y., Liu Y.-C., Hong T.-H., Yu S.-L., Chen J.J. (2015). Opposite effects of M1 and M2 macrophage subtypes on lung cancer progression. Sci. Rep..

[56] Hanaka T., Kido T., Noguchi S., Yamada S., Noguchi H., Guo X., Nawata A., Wang K.-Y., Oda K., Takaki T. (2019). The overexpression of peroxiredoxin-4 affects the progression of idiopathic pulmonary fibrosis. BMC Pulm. Med..

[57] Abbas K., Breton J., Planson A.-G., Bouton C., Bignon J., Seguin C., Riquier S., Toledano M.B., Drapier J.-C. (2011). Nitric oxide activates an Nrf2/sulfiredoxin antioxidant pathway in macrophages. Free. Radic. Biol. Med..

[58] Mills C.D., Ley K. (2014). M1 and M2 macrophages: The chicken and the egg of immunity. J. Innate Immun..

[59] Kim J., Lee G.-R., Kim H., Jo Y.-J., Hong S.-E., Lee J., Lee H.I., Jang Y.-S., Oh S.-H., Lee H.J. (2016). Effective killing of cancer cells and regression of tumor growth by K27 targeting sulfiredoxin. Free. Radic. Biol. Med..

[60] Ye Q., Zhang Y., Cao Y., Wang X., Guo Y., Chen J., Horn J., Ponomareva L.V., Chaiswing L., Shaaban K.A. (2019). Frenolicin B targets peroxiredoxin 1 and glutaredoxin 3 to trigger ROS/4E-BP1-mediated antitumor effects. Cell Chem. Biol..

[61] Lee T.H., Jin J.-O., Yu K.J., Kim H.S., Lee P.C.-W. (2019). Inhibition of peroxiredoxin 2 suppresses Wnt/β-catenin signaling in gastric cancer. Biochem. Biophys. Res. Commun..

[62] Ding N., Jiang H., Thapa P., Hao Y., Alshahrani A., Allison D., Izumi T., Rangnekar V.M., Liu X., Wei Q. (2022). Peroxiredoxin IV plays a critical role in cancer cell growth and radioresistance through the activation of the Akt/GSK3 signaling pathways. J. Biol. Chem..

[63] Abbasi A., Corpeleijn E., Postmus D., Gansevoort R.T., de Jong P.E., Gans R.O., Struck J., Schulte J., Hillege H.L., van der Harst P. (2012). Peroxiredoxin 4, A novel circulating biomarker for oxidative stress and the risk of incident cardiovascular disease and all-cause mortality. J. Am. Heart Assoc..

[64] Tiedemann K., Hussein O., Sadvakassova G., Guo Y., Siegel P.M., Komarova S.V. (2009). Breast cancer-derived factors stimulate osteoclastogenesis through the Ca2+/protein kinase C and transforming growth factor-β/MAPK signaling pathways. J. Biol. Chem..

[65] Rafiei S., Komarova S.V. (2013). Molecular signaling pathways mediating osteoclastogenesis induced by prostate cancer cells. BMC Cancer.

[66] Rafiei S., Tiedemann K., Tabariès S., Siegel P.M., Komarova S.V. (2015). Peroxiredoxin 4: A novel secreted mediator of cancer induced osteoclastogenesis. Cancer Lett..

