# Supplementary material for: Critical Role of the Sulfiredoxin-Peroxiredoxin IV Axis in Urethane-Induced Non-Small Cell Lung Cancer
Source: Antioxidants (Basel). 2023 Feb 3;12(2):367. doi: 10.3390/antiox12020367 (PMC9951953; doi:10.3390/antiox12020367)
Supplement: Supplementary file 1 [file antioxidants-12-00367-s001.zip › antioxidants-2186441-supplementary.pdf]

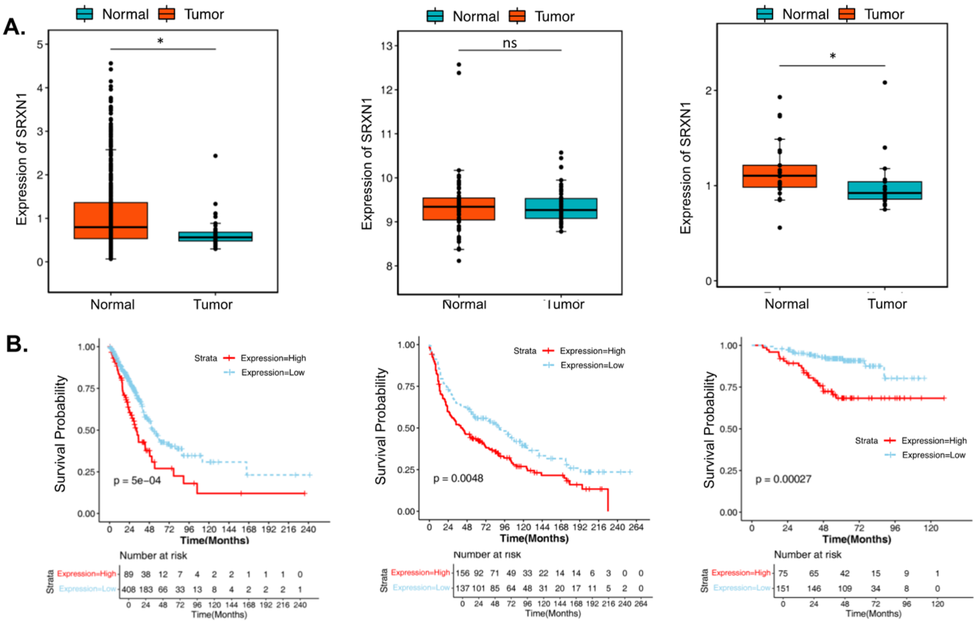

Figure S1. SRXN1 is up-regulated in lung adenocarcinoma, and negatively related to prognosis.(A) The expression level of SRXN1 between LUAD tumor and normal groups from TCGA-LUAD, GSE19804, and GSE27262 datasets.(B) K-M survival curves of SRXN1 expression in LUAD patients. (*, $p < 0.05$; ns, $p \geq 0.05$).

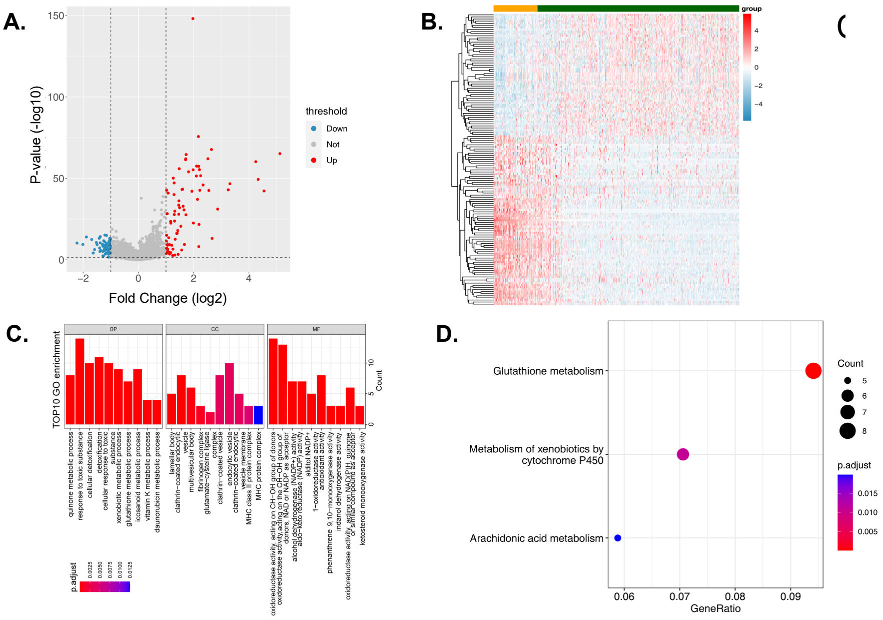

Figure S2. Identification and exploration of DEGs between the high- and low-expression groups of SRXN1.(A,B). Volcano plot and heatmap of DEGs in LUAD cohort (adj.P.Value < 0.05 and |log2fold change| >1).(C,D). Bubble charts of the GO) and KEGG enrichment analyses.

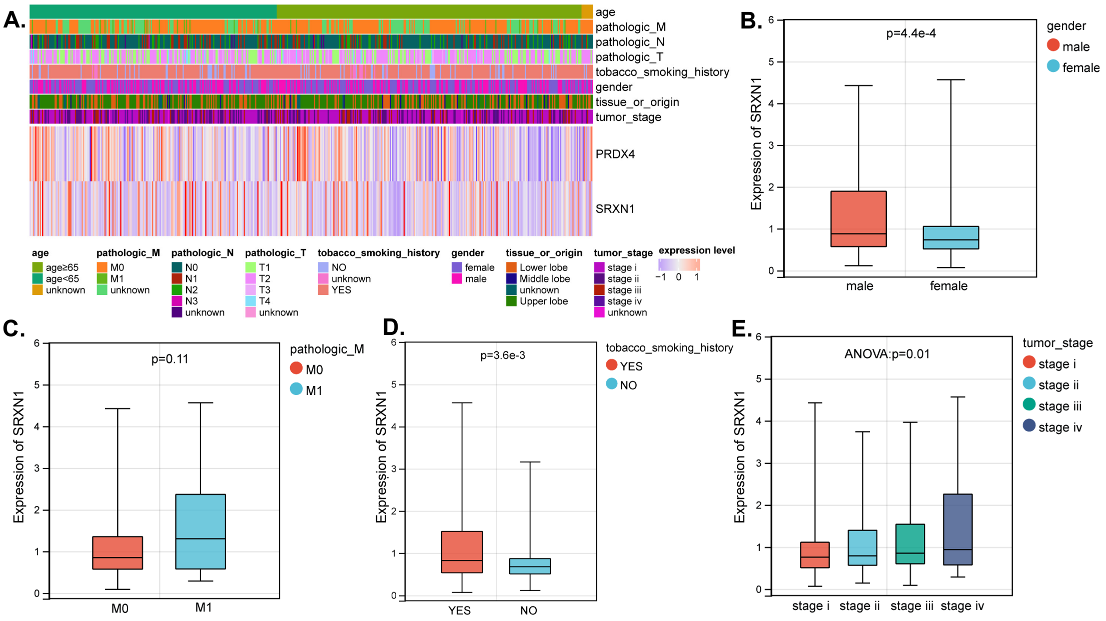

Figure S3. The correlation analysis of SRXN1 expression and different clinical features.(A). SRXN1 and Prx4 expression in LUAD cohort with different clinical features.(B). Boxplot of SRXN1 expression in LUAD cohort with different gender, pathologic M, tobacco smoking history, and tumor stage.

Table S1. Clinical characteristic of LUAD patients between the high- and low-expression groups of SRXN1.

FEATURES	HIGH SRXN1 (N=89)	LOW SRXN1(N=408)	P.VALUE
age			0.9796
age<65	40	178	
age≥65	48	221	
pathologic_M			0.002377
M0	59	272	
M1	11	13	
pathologic_N			0.1122
N0	49	272	
N1	22	72	
N2	17	52	
N3	0	2	
pathologic_T			0.9074
T1	30	136	
T2	46	221	
T3	8	35	
T4	4	14	
tobacco_smoking_history			0.02913
NO	6	65	
YES	83	329	

gender			3.342e-05
female	30	239	
male	59	169	
tissue_or_origin			0.6106
Lower lobe	28	140	
Middle lobe	2	19	
Upper lobe	54	237	
tumor_stage			0.003266
stage i	37	230	
stage ii	22	96	
stage iii	17	63	
stage iv	11	14	
